# Fever Through a Jaundiced Eye

**DOI:** 10.1177/2324709614533513

**Published:** 2014-04-29

**Authors:** Christina C. Kennelly, Chelsey A. Petz, Don C. Rockey

**Affiliations:** 1 Medical University of South Carolina, Charleston, SC, USA

**Keywords:** liver, abscess, infection, pyogenic

## Abstract

Pyogenic liver abscess (PLA) is an important clinical entity to consider in a patient with fever and abdominal pain. Previously, the condition was difficult to diagnose and treat, but with the introduction of widely available and reliable imaging techniques, its diagnosis has become more straightforward. Although uncommon, PLA should especially be considered in the differential diagnosis for patients with specific predisposing conditions such as underlying biliary tract disease, whether as a result of chronic inflammatory disease or malignancy. The introduction of percutaneous drainage has revolutionized the management of PLA, and thus, this disease has become largely correctable.

## Introduction

When originally described, pyogenic liver abscess (PLA) was typically a consequence of acute appendicitis with pylephlebitis and was associated with a high mortality.^1^ Furthermore, in this early era, surgery was considered to be the primary therapeutic option.^1^ Today, the etiology, bacteriology, and management of this condition have dramatically changed. This has resulted in improved clinical outcomes, making it a critically important clinical condition to consider in certain patient populations.

## Case Presentation

A 47-year-old male with a past medical history of primary sclerosing cholangitis and hypertension was admitted with progressive fatigue, fever, chills, and night sweats for the past 4 to 5 weeks. His fevers reached a temperature maximum of 102°F at home. He also had some exertional dyspnea and occasional cough but denied shortness of breath or chest pain. He reported an approximate weight loss of 26 pounds in the past 6 months. Approximately 10 days before presentation, he was seen by his primary care physician and diagnosed with pharyngitis and was started on azithromycin and levofloxacin but did not improve. Additionally, he admitted to small amounts of bright red blood in his stool over the past several days but denied melena or hematemesis. He had been taking several ibuprofens over the past several days for low back pain.

Six months earlier, the patient presented with decreased energy and was found to have primary sclerosing cholangitis. He had a colonoscopy demonstrating mild patchy erythema in the transverse colon and terminal ileum and external hemorrhoids. Biopsies of the terminal ileum were normal, but biopsies of the colon revealed changes consistent with focal colitis with eosinophilic infiltration. He typically reported 1 to 2 formed bowel movements a day that were only occasionally loose without blood or mucus. At the time of admission he was taking ferrous sulfate, vitamin C, vitamin B_12_, a multivitamin, cetirizine, valsartan/hydrochlorothiazide, fluticasone, and the levofloxacin. He had completed the course of azithromycin. He had a previous cholecystectomy. He had a past history of tobacco use, and he drank occasional alcohol in his 20s but none recently. His family history was unremarkable.

He was jaundiced, and he appeared fatigued and was thin. His temperature was 37.3°C, blood pressure 117/73 mm Hg, and pulse 73 beats per minute. The respiratory rate was 16, and the oxygen saturation was 97% on ambient air. He was not orthostatic. He had scleral icterus and moist mucosa. Bilateral lower lobe rhonchi were auscultated. The abdomen was soft, nontender to palpation, and not distended. No hepatosplenomegaly was palpated. Digital rectal exam demonstrated an external hemorrhoid without blood or stool in the vault; his prostate was mildly enlarged. The remainder of the exam was normal.

The patient’s white blood cell count was 11 000 mm^−3^. An automated differential revealed 68% neutrophils, 21% lymphocytes, 5% monocytes, and 6.6% eosinophils. The hemoglobin level was 6.3 g/dL, and the platelet count was 382 000 mm^−3^. The mean corpuscular volume was 91 fL. His total bilirubin was 7.7 mg/dL, direct bilirubin 4.1 µg/mL, aspartate aminotransferase 54 U/L (normal value = 12-38 U/L), alanine aminotransferase 51 U/L (normal value = 10-45 U/L), alkaline phosphatase 703 U/L (normal value = 25-100 U/L), total protein 8 g/dL, and albumin 1.8 g/dL. The international normalized ratio was 1.3, and prothrombin time was 16.6 seconds. His iron was 47 µg/dL, total iron binding capacity 213 µg/dL, ferritin 341 ng/mL, and transferrin 152 mg/dL. His reticulocyte count was 6.11%. The haptoglobin was 306 mg/dL, and lactate dehydrogenase was 364 U/L.

**Figure 1.**
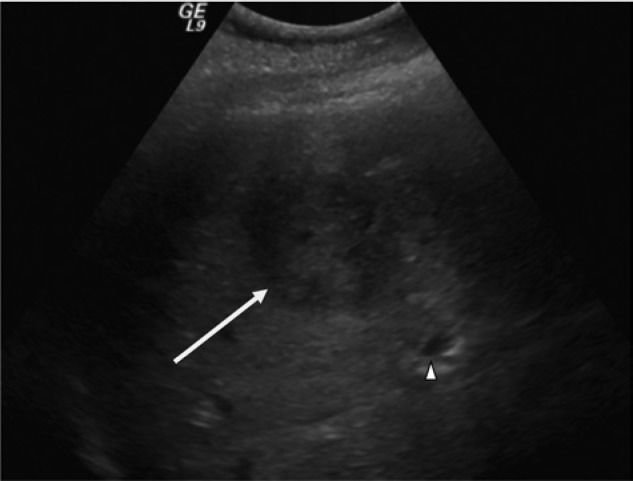
Right upper quadrant ultrasound. A space-occupying lesion (white arrow), consistent with a pyogenic liver abscess, is visible in the right lobe of the liver. Dilated intrahepatic ducts (white arrowhead) can also be seen.

**Figure 2.**
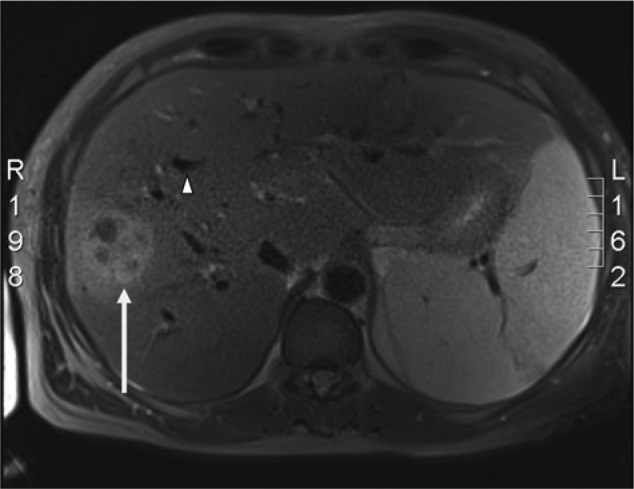
Liver magnetic resonance cholangiopancreatography. The space-occupying lesion seen on ultrasound (white arrow), consistent with a pyogenic liver abscess, can be seen in the right lobe of the liver; multiple dilated intrahepatic biliary ducts (white arrow head) are also present.

Abdominal ultrasound revealed the common bile duct to measure 7 mm, and his intrahepatic ducts were noted to be dilated and irregular. In addition, there were 2 solid appearing lesions in close proximity to each other in the right lobe of the liver, one measuring 4 cm and the other 6 cm in diameter (see Figure 1). Magnetic resonance cholangiopancreatography revealed a 10.2 × 6.2 centimeter peripherally enhancing mass with septations in the right hepatic lobe (see Figure 2).

**Figure 3.**
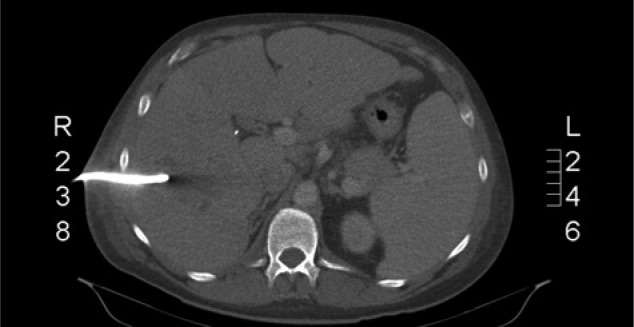
Computed tomography–guided drain placement of pyogenic liver abscess with. A catheter was placed in the space-occupying lesion seen above by ultrasound and magnetic resonance cholangiopancreatography. This yielded purulent fluid. A drain was placed, which can be seen traversing the abdominal wall in the region occupied by the pyogenic liver abscess.

**Figure 4.**
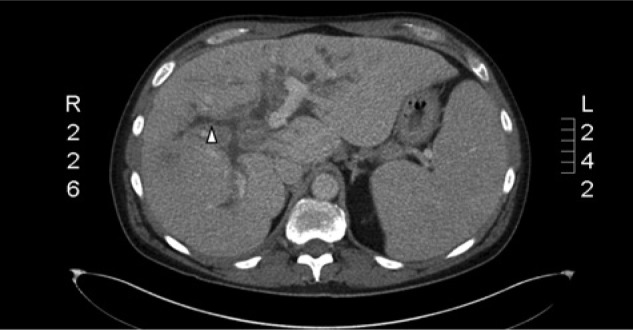
Liver computed tomography after drain placement. While dilated intrahepatic biliary ducts (white arrow head) persist throughout the liver, the liver abscess is no longer visible.

On admission, the patient was empirically treated with vancomycin and piperacillin/tazobactam for possible cholangitis and/or liver abscess. The patient underwent computed tomography–guided aspiration of the lesion and purulent fluid was noted; a drain was placed (see Figure 3), and culture of the fluid grew *Streptococcus milleri*. After susceptibilities showed that the organism was susceptible to ceftriaxone, this was continued for 1 month intravenously, followed by 2 weeks of oral amoxicillin. Repeat abdominal imaging 1 month after drain placement demonstrated an ill-defined area of low attenuation in the right hepatic lobe with no evidence of a drainable fluid collection at which point the drain was removed (Figure 4). Although dilated intrahepatic ducts were still noted, the patient felt well after completion of his antibiotic courses and drain removal.

## Discussion

Our patient’s presentation was classic for PLA. Historically, PLAs were a complication of inadequately treated intra-abdominal infections, namely, pylephlebitis secondary to appendicitis.^1^ In the current era, biliary tract disease has emerged as the most common predisposing factor for PLA, as was the case in our patient.^2-4^ PLA caused by biliary tract disease may be caused by either malignant or benign biliary tract disease, the latter most often gallstone disease, but also potentially diseases such as primary sclerosing cholangitis (PSC). Importantly, PSC is often associated with inflammatory bowel disease, and the latter is typical in patients with PSC and associated with PLA. Our patient had a colonoscopy demonstrating some trivial colonic inflammation but did not have definitive evidence of inflammatory bowel disease. Other etiologies of PLA are based on the route of extension of infection: from blunt or penetrating abdominal trauma, by direct extension, or by hematogenous spread. Importantly, a substantial number of patients are found to have no predisposing identifiable source.^2,3,5^ Additionally, complications associated with interventional procedures and surgical techniques have resulted in PLAs.^6-8^

Patients with PLA typically present with fever and abdominal pain.^2,5,9^ Other nonspecific symptoms including nausea, vomiting, anorexia, weight loss, and malaise are also common.^2,5,10^ Our patient presented with fever, fatigue, and night sweats over 4 to 5 weeks, a classic presentation for patients with PLA. However, we would emphasize that because these symptoms are nonspecific it is important to have a high degree of suspicion for PLA as a potential diagnosis in patients with a history of underlying biliary disease. One other point is that differentiation of PLA and amebic liver abscess (AMA) is often difficult and requires a combination of historical, clinical, and laboratory information to make an accurate diagnosis.^11^ Fortunately, amebic serology in patients with AMA is very sensitive and specific and is thus very helpful in this clinical setting.

As the underlying etiology of PLAs have evolved, so too has the microbiology of the disease. *Escherichia coli, Klebsiella*, and streptococcal species are the organisms most commonly isolated; however, PLAs are frequently polymicrobial.^2,5^ Studies differ on the most common organism isolated, and it usually determined by the underlying etiology of the liver abscess and the region of the study. Our patient had *Streptococcus milleri* isolated from his PLA; interestingly, this organism has been isolated in a high proportion of cases of PLA complicating inflammatory bowel disease.^12^

Antibiotics should be employed as soon as the diagnosis of PLA is suspected. The suspected origin of the PLA should be considered when choosing empiric antibiotics. Abscesses that arise in patients with biliary disease have been associated with gram negative bacilli and initial antibiotics should include coverage of these organisms.^13^ Colonic or pelvic sources of PLAs are more commonly due to anaerobes and this should be considered with initial antibiotic selection.^13^ Some advocate adding metronidazole to initial therapy for liver abscesses to cover for anaerobes; additionally, in the setting of an abscess of unclear origin, metronidazole also covers AMA. Thus, this approach should be considered in patients with risk factors (recently traveled to an endemic area or immunosuppressed) for AMA.^11,13^ Once a causative organism is identified and susceptibility profiles obtained, antibiotics may be tailored to the isolated organism.

Management of PLA is primarily by percutaneous drainage, and current evidence suggests excellent outcomes with this approach.^14^ Although surgery had been the primary mode of treatment for many years, percutaneous drainage and aspiration have been found to confer similarly high success rates as an open surgical approach with the advantages of being a minimally invasive approach.^2,14^ Thus, surgery is now reserved for those patients who have failed percutaneous drainage, those who require surgery for the underlying problem, and some patients with large, loculated, or multiple abscesses.^5,15,16^ Continuous drainage is typically recommended, but aspiration alone may be effective in some patients. For some small abscesses (typically less than 3 cm), and certain other patients, antibiotics alone may be sufficient for therapy, but this should be reserved for select cases. If this approach is taken, prolonged (several months) antibiotics are recommended (and even in situations where drainage is successful, an extended course of multiple weeks should be given). As in our patient, if cultures grow specific organisms, antibiotics may be tailored accordingly, although it should be emphasized that anaerobes may not be isolated and that coverage that includes anaerobes is typically best.

A final point in this case has to do with management of this patient’s biliary tract disease. While there was not obvious evidence of malignancy, and it was presumed that his underlying primary sclerosing cholangitis was the cause of the PLA, it should be emphasized that in most patients a thorough search for underlying pathology should be sought. One of the most dreaded complications of primary sclerosing cholangitis is cholangiocarcinoma, and this must always be considered in patients such as that presented here.

## Conclusion

In summary, PLA is an important and, if left untreated, uniformly fatal disorder. However, with appropriate therapy, the prognosis is excellent. Changes in etiology, imaging, and management of this disease have dramatically altered the outcomes in these patients. Given the nonspecific presentation of the disease, clinicians should have a high index of suspicion for it, especially in the setting of underlying predisposing diseases.
